# Agony resulting from cultural practices of canine bud extraction among children under five years in selected slums of Makindye: a cross sectional study

**DOI:** 10.1186/s12903-018-0599-y

**Published:** 2018-08-07

**Authors:** Fiona Atim, Teddy Nagaddya, Florence Nakaggwa, Mary Gorrethy N-Mboowa, Peter Kirabira, John Charles Okiria

**Affiliations:** 1 Clarke International University (CIU) formerly International Health Sciences University (IHSU), Plot 4686, St. Barnabas Road Kisugu-Namuwongo, P.O Box 7782, Kampala, Uganda; 2 St. Lawrence University, Kampala, Uganda

**Keywords:** Canine bud extraction, Cultural practices, Children slums

## Abstract

**Background:**

Canine Bud Extraction (CBE) is a process of removing or gouging children’s healthy canine tooth buds embedded underneath the gum using traditional unsterilized tools. The practice of CBE commonly known as false teeth removal continues to be an adopted cultural intervention of choice, in the prevention of morbidity and mortality from common childhood illnesses. However, it is a practice against the rights of the children with serious consequences. While CBE is associated with the perceived myth of curative gains, the agony emanating from the cultural practice exposes children to ill-health conditions such as dehydration, malnutrition, blood-borne diseases like HIV/AIDs, septicemia, fever and death. This research sought to understand the factors underpinning the practice of CBE among urban slum dwellers.

**Method:**

A cross-sectional study was conducted from five randomly selected slums in Makindye division; 298 household heads or guardians with children below 5 years, who had ever suffered from false teeth were interviewed. The variables measured included guardians’ socio-demographic profiles, determinants of CBE, common childhood illnesses assumed to be treated with CBE and the reported side-effects associated with the practice.

**Results:**

Of the 298 respondents with children who had ever suffered from “false teeth” interviewed, 56.7% had two or more children below 5 years and 31.9% were from the central region. The proportion of households practicing CBE was 90.3%; 69.8% of the caretakers mentioned that it was done by traditional healers and for 12.1% by trained health workers (dentists). Number of children (OR = 2.8, 95% CI: 1.1–7.2) and the belief that CBE is bad (OR = 0.1, 95% CI: < 0.001, *p* < 0.001) had a statistically significant association with CBE. Additionally, number of children (χ2 = 4.9, *p* = 0.027) and 2 sets of beliefs (CBE treats diarrhea (χ2 = 12.8, *p* = 0.0017) and CBE treats fever (χ2 = 15.1, *p* = 0.0005) were independent predictors of CBE practice. A total of 55.7% respondents knew that there were side effects to CBE and 31% mentioned death as one of them.

**Conclusion:**

The high proportion of households practicing CBE from this study ought to awaken the perception that the practice is ancient. CBE in this community as the study suggests was strongly driven by myths. The strong belief that CBE is bad provides an opportunity for concerted effort by primary health care providers, policy makers and the community to demystify the myths associated with false teeth and the gains of CBE.

## Background

Canine Bud Extraction is a process of removing or gouging children’s healthy canine tooth buds embedded underneath the gum using traditional unsterilized tools. Many terms or names are used to mean Canine Bud Extraction which include; Infant Oral Mutilation (IOM), germectomy, Tooth Bud Extraction (TBE), and Deciduous Canine Tooth Bud Extraction (DCBE) [1]. Canines are also called eye teeth or cuspids used to ripe and tear tough foods and create bifurcation between the front teeth and back teeth [2] ill health conditions associated with CBE constitute greater than (75%) causes of under-five mortality in Sub-Saharan Africa with Uganda taking the lead. However, it is a practice against the rights of the children leading to serious health consequences [3] . The practice of CBE is known to have emanated from Northern Uganda [4] as the remedy for “false teeth”. The concept of false teeth is believed to be brought about by traditional healers who in their attempts to find the causes of diarrheal diseases and fevers in children found that un-erupted canines together with tooth buds was an abnormal occurrence and were identifies as the causes of diarrhea, vomiting and fever in children, and that extracting them would relieve the children from those diseases [5]. Literature available indicates that CBE was rampant in the 1980’s and 1990’s [1, 6–8]. However, current literature shows that the practice is now common among African migrants to western countries [1, 5].

### History of canine bud extraction

The practice of CBE is a superstitious belief whose origin is unknown. CBE is evident in most of the African and Sub-Saharan African countries with the first cases being recorded as early as 1932 among the Nilotic tribes [1] Some of the countries known to practice CBE include; Chad, Kenya, Somalia, Tanzania, Rwanda, Sudan, Congo, Uganda and the United Kingdom because of immigration [4–7, 9–11] other western countries where CBE was reported include; Sweden, Norway, France, New Zealand and Australia.

A survey conducted in 2013 among offspring of Ethiopian immigrants indicated that the prevalence of missing canines and poor dentition (poor teeth formation) is higher among emigrants from Ethiopia than those from Israel living in the same low socio-economic status [12].

Reports received in 2009 from DR. Congo former Zaire showed that CBE was common among people from Aru and Kivu areas and this practice was introduced from Uganda during Idi Amin’s regime when Ugandan refugees entered Congo; the same experience was seen in Burundi and Rwanda [6]. A report in 2013 [12] indicates that the prevalence of CBE varied in African countries with Sudan having 22%, Tanzania 37.4%, and Uganda 17.2% among other African countries.

Studies conducted in Uganda show high prevalence of CBE. A study by Tirwomwe [13] in six districts of Uganda (Kabale, Mbale, Kampala, Arua, Gulu and Masindi) indicates an overall prevalence of 29.3%. However, findings of another study indicate that the prevalence of CBE was high in Northern Uganda with 55.1% in Gulu and 41.0% in Arua; in Masindi, the prevalence was at 36.1%, Kabale 21.8% and Mbale 22.5% [1, 5, 14]. Furthermore, a study carried out in Western Uganda [13] among children under 5 years, indicated that false teeth cause diarrhea, acute respiratory infection, fever and loss of appetite. Other key signs and symptoms of the same were believed to be treated with dental surgery which is done by traditional healers. It also suggested that conditions associated with false teeth which leads to CBE constitutes greater than (75%) causes of under-five mortality in the country and other Sub- Saharan African and East African countries; the practice is also associated with the fact that most health seeking behavior for the condition is directed to local traditional healers present in the local communities.

### Study site context

The study was conducted in the slum areas of Makindye Division because most of the studies conducted on CBE have mainly been in hospitals, villages, Peri-urban settings and urban areas. The slum areas were also selected by the fact that they harbor the urban poor from different regions from Uganda and constitute different nationalities.

## Methods

A cross sectional study design was used where 298 respondents consented before being interviewed; both qualitative and quantitative methods of data collection were used to obtain information regarding the above stated study. A researcher/interviewer administered questionnaire was used and translation to the local language made to accommodate those who were not able to communicate in English. The study was done in households with children under the age of 5 years who had ever suffered from “false teeth” living in the five randomly selected slums within (Namwongo Zone A, Kisugu central Zone, Kibuli Kisaga, Kikubamutwe, Wabigalo project Zone) known to be characterized by slums in Makindye Division, Kampala. These are characterized by urban neighborhoods of the lowest socio-economic level populated by migrants in to the city within informal settlements. The decision about CBE is not necessarily made by parents, but rather caretakers of children under the age of 5 years.

The researcher used stratified sampling technique using probability proportionate to population size to determine the number of households to be included in the study. Probability proportionate to sample size reduces on the chances of selecting more members from a larger group as opposed to a smaller group. This was used since the selected slums did not have equal number of households and household heads.

The study area was first stratified by village, eligible population in each slum by village determined, and the number of household. Proportionate number of household was determined, and simple random sampling used to select the households to be included in the study. For each household visited, a household head/caregiver found with a child below 5 years was included in the study and where there was no eligible participant, the next household found with the eligible participant was considered. The households had to have children below 5 years because the practice of CBE is common among children below the age of 5 years.

Prior to data collection, clearance to collect data was sought from the University Research Scientific Committee of International Health Sciences University and the respective heads of the selected villages. Informed consent was also sought from the study participants where participation was voluntary and the choice to withdraw from the study at any point in time observed. Data collected was cleaned on a daily basis to promote good quality data.

### Participants’ demographic characteristics (Table 1)

A total of 298 study participants were enrolled for the study with Namuwongo 102 (34.2%) contributing the highest number of respondents and Wabigalo 28(9.4%) the least. Most of the study participants were female 262 (87.9%), aged 26–31 years 115(38.6%), had primary level education 108(36.2%), were catholic 118(39.6%) and married 22(76.2%); 188(63.1%) were employed and 169(56.7%) had two or more children below 5 years old. The highest proportion of the respondents was from the central region 95 (31.9%).

**Table 1:** Study Participants’ characteristics

Characteristic	Frequency (*N* = 298)	Percentage	Unadjusted OR (95% CI)	*P*-value
Zone				
Namuwongo	102	34.2	1	
Kisugu central	88	29.5	0.4 (0.2–1.1)	0.089
KibuliKisaga	48	16.1	2.0 (0.4–9.6)	0.407
Kikubamutwe	32	10.7	1.3 (0.3–6.3)	0.765
Wabigalo	28	9.4	0.7 (0.2–2.9)	0.630
Sex				
Male	36	12.1	1	
Female	262	87.9	0.5 (0.1–2.3)	0.376
Age				
14–19	6	2.0	1	
20–25	59	19.8	1.3 (0.1–12.4)	0.834
26–31	115	38.6	1.7 (0.2–15.9)	0.635
32–38	75	25.2	2.8 (0.3–28.8)	0.387
39+	43	14.4	2.7 (0.2–30.8)	0.432
Education level				
None	52	17.5	1	
Primary	108	36.2	0.4 (0.1–1.7)	0.186
Secondary	106	35.6	0.3 (0.1–1.6)	0.178
Tertiary	32	10.7	0.2 (0.0–1.2)	0.078
Religion				
Catholic	118	39.6	1	
Protestant	90	30.2	0.5 (0.2–1.4)	0.183
Muslim	58	19.5	0.5 (0.2–1.4)	0.165
Other	32	10.7	0.3 (0.1–1.2)	0.084
Marital status				
Single	36	12.1	1	
Married	227	76.2	1.1 (0.3–3.2)	0.922
Sep/di*v*/*w*id^1^	35	11.7	4.3 (0.5–40.1)	0.207
Employment				
Unemployed	110	36.9	1	
Employed	188	63.1	1.2 (0.6–2.7)	0.600
Tribe				
Western	57	19.1	1	
Northern	61	20.5	4.1 (0.8–20.8)	0.081
Central	95	31.9	0.7 (0.3–2.0)	0.553
Eastern	64	21.5	1.7 (0.5–5.5)	0.415
Other	21	7.1	–	–
No of children				
One	129	43.3	1	
Two or more	169	56.7	2.3 (1.1–5.1)	0.036^a^

^a^Statistically significant at *p* = 0.05, 1 *sep/div/wid* separated/widow/divorced

### Relationship between canine bud extraction and socioeconomic factors

From Table 1, the odds of CBE were lower among females compared to male respondents (OR:0.5, 95% CI:0.1–2.3), primary (OR:0.4, 95% CI:0.1–1.7), secondary (OR:0.3, 95% CI:0.1–1.6) and tertiary (OR:0.2, 95% CI:0.0–1.2) education levels compared to no education, all listed religions compared to catholic religion, and in central (OR:0.7, 95% CI:0.3–2.0) compared to western tribes. The odds of CBE were higher in all age groups compared to 14–19 years old (almost 3 times higher in age groups 32–38 years (OR:2.8, 95% CI:0.3–28.8) and 39 years or older (OR:2.7, 95% CI:0.2–30.8)), married and those separated, widowed or divorced compared to single respondents, 20% higher among those employed (OR:1.2, 95% CI:0.6–2.7) compared to unemployed respondents, 2.3 times higher among respondents who had 2 or more children below 5 years compared to those who only had 1 child (OR:2.3, 95% CI:1.1–5.1) and 4 times higher among respondents of northern tribes compared to Western tribes (OR: 4.1 95% CI: 0.8–20.8); All foreign tribes had practiced CBE. Only number of children below 5 years was significantly associated with CBE (*p*-value = 0.036).

### Proportion of households that practiced CBE

Up to 269 (90.3%) of the respondents with children who had ever suffered from “false teeth” reported subjecting their children to canine bud extraction (CBE); only 29 (9.7%) respondents reported not subjecting their children to canine bud extraction. Participants were further asked where they sought the canine bud extraction services and a majority (69.8%) mentioned that they had gone to traditional healers; 12.1% of the study participants had their children’s canine buds extracted by a health worker. The children had their teeth extracted at 3 years (48%), 2 years (33.2%), 4 years (12.4%), 1 year (6%) and 5 years (0.3%).

With regards to instruments used during CBE, 82 (27.5%) reported that bicycle spokes were used, 71 (23.8%) said that surgical/razor blades were used, 54 (18.1%) mentioned nails, and 33 (11.1%) mentioned knives; 19.5% did not know what had been used during the CBE process, 56.9% of these had gone to traditional healers.

Those who reported not performing canine bud extraction were asked what they had done about the false teeth; 24 (82.8%) of them said that the children’s teeth had been scrubbed while 5 (17.2%) had sought medical attention. Among the things used for scrubbing were herbal medicines (used by 21 respondents), honey and garlic (used by 3 respondents).

### Knowledge

All research participants reported having ever heard about CBE. The sources of information mentioned by the study participants on the CBE practice included 22 (7.4%) from traditional healers, 10 (3.4%) from health workers, 24 (8.1% from spouses, 50 (16.8%) from friends 71 (23.8%) from neighbors and 121 (40.6%) from other relatives (see Table 2 for more details).

**Table 2:** Multivariate analysis of relationship between CBE and knowledge and perception factors using stepwise selection

Characteristic	Total *N* = 298	%	Unadjusted OR (95% CI)	*P*-value	Adjusted OR (CI)^b^	*P*-value
CBE Information source						
Friend	50	16.8	1			
Neighbor	71	23.8	1.3 (0.4–3.8)	0.654		
Health worker	10	3.4	1.5 (0.2–13.4)	0.735		
Traditional healer	22	7.4	1.6 (0.3–8.5)	0.565		
Spouse	24	8.1	1.1 (0.3–4.9)	0.860		
Relative	121	40.6	2.3 (0.9–6.7)	0.128		
Know side effects						
No	136	45.6	1			
Yes	162	54.4	0.4 (0.2–1.0)	0.062		
CBE leads to poor dentition						
Disagree	45	15.1	1			
Agree	170	57.1	0.6 (0.2–2.3)	0.497		
Don’t know	83	27.8	0.6 (0.2–2.3)	0.443		
CBE treats fever						
Disagree	11	3.7	1			
Agree	275	92.3	17.1 (4.7–61.6)	< 0.001^a^		
Don’t know	12	4.0	1.7 (0.3–8.8)	0.538		
CBE treats diarrhea						
Disagree	11	3.7	1		1	
Agree	281	94.3	7.5 (2.0–27.6)	0.003^a^	1.2 (0.1–19.6)	0.875
Don’t know	6	2.0	0.1 (0.0–1.4)	0.086	0.02 (0.0–1.0)	0.051
CBE treats malaria						
Disagree	15	5.0	1	1	1	
Agree	186	62.4	4.8 (1.4–17.3)	0.015^a^	2.8 (0.2–44.5)	0.461
Don’t know	97	32.6	2.6 (0.7–9.4)	0.152	2.7 (0.2–48.3)	0.497
CBE treats cough						
Disagree	49	16.4	1		1	
Agree	111	37.3	2.4 (0.7–8.7)	0.181	1.3 (0.3–6.5)	0.766
Don’t know	138	46.3	0.7 (0.3–2.0)	0.523	0.5 (0.1–2.1)	0.347
CBE treats vomiting						
Disagree	14	4.7	1		1	
Agree	195	65.4	3.8 (0.9–15.4)	0.060	3.7 (0.5–27.1)	0.192
Don’t know	89	29.9	1.6 (0.4–6.5)	0.515	2.6 (0.4–18.4)	0.325
Consider CBE bad						
No	181	60.7	1		1	
Yes	117	39.3	0.1 (0.1–0.4)	< 0.001^a^	0.1 (0.0–0.4)	< 0.001^a^
No of children						
One	129	43.3	1		1	
Two or more	169	56.7	2.3 (1.1–5.1)	0.036^a^	2.8 (1.1–7.2)	0.038^a^

^a^Statistically significant at *p* = 0.05, ^b^Adjusted for diarrhea treats CBE, malaria treats CBE, vomiting treats CBE, consider CBE bad and number of children

More than half of the research participants (55.7%) reported knowing the side effects of canine bud extraction whereas 44.3% said they did not know of any side effects of CBE; of those who reported side effects, 17 (10.4%) mentioned included death, 51 (31.3%) poor dentition (which meant funny shaped teeth and overcrowding of teeth in the mouth by the participants), 26 (16%) failure to develop canine teeth, 34 (20.9%) exposure to infections, 16 (9.8%) severe pain and 19 (11.7%) over bleeding. Respondents were asked to verify several statements regarding CBE; 94.3, 92.3, 65.4, 62.4 and 37.3% agreed with the statements “CBE treats diarrhea”, “CBE treats fever”, “CBE treats vomiting”, “CBE treats malaria” and “CBE treats cough” respectively; 57.15% agreed with the statement “CBE leads to poor dentition” (see Table 2 for more details).

Concerning knowledge about the place where CBE is done, 69.8% mentioned traditional healers, 15.1% community elders, 12.1% health workers and 0.3% had the practice done by themselves (self).

### Cultural beliefs

In all, 178 (59.7%) respondents reported that they had cultural beliefs related to false teeth while 120 (40.3%) said that they did not have such beliefs. Of those who reported the existence of cultural beliefs, 120 (66.3%) did not know the beliefs, 13 (7.3%) said that false teeth were caused by germs, 36 (20.2%) claimed that they are caused by “stepping on false teeth when pregnant”, and 8 (5.1%) indicated that it was a family disease. Respondents were further asked whether the canine bud extraction practice was a taboo in their cultures; 267 (89.6%) said that it was not and only 31 (10.4%) said that it was. The reasons given for why CBE practice was a taboo were “it is a foreign cultural practice” mentioned by 10 respondents and “CBE kills” mentioned by 6 respondents and “it is associated with witchcraft” mentioned by 15 respondents.

### Perception

Most of the respondents 173, (58.1%) perceived CBE to be a good practice, yet 22.1% thought that it was a bad practice, 15.1% thought that it should be banned while 4.7% believed that it was a very painful practice.

### Multivariate analysis of relationship between CBE and knowledge and perception factors

From Table 2, the odds of the CBE practice were 60% lower among respondents who knew the side effects (OR:0.4, 95% CI:0.2–1.0) compared to those who did not know, 40% lower among those who believed that CBE causes poor dentition (OR:0.6, 95% CI:0.2–2.3) compared to those who disagreed with this, 90% lower among those who considered CBE a bad practice (OR:0.1, 95% CI:0.1–0.4) compared to those who did not, and 90 and 30% lower among respondents who did not know whether CBE caused diarrhea (OR:0.1 95% CI:0.0–1.4) and cough (OR:, 95% CI:0.3–2.0) respectively compared to those who disagree with the statement. Furthermore, the odds of CBE practice were higher among those who believed that CBE treats fever (OR:17.1, 95% CI:4.7–61.6), diarrhea (OR:7.5, 95% CI:2.0–27.6), malaria (OR:4.8, 95% CI:1.4–17.3), cough (OR:2.4, 95% CI:0.7–8.7) and vomiting (OR:3.8, 95% CI:0.9–15.4) compared to those who disagreed with these notions. After adjusting for the effect of diarrhea treats CBE, malaria treats CBE, vomiting treats CBE, consider CBE bad and number of children, the odds of CBE practice remained higher in these groups compared to those who disagreed. Only perception of CBE as a bad practice and a number of children remained associated with CBE.

Additionally, belief that CBE treats diarrhea (χ^2^ = 12.8, *p* = 0.0017), number of children (χ^2^ = 4.9, *p* = 0.027) and CBE treats fever (χ^2^ = 15.1, *p* = 0.0005) remained independent predictors of CBE practice even after multivariate analysis.

## Discussion

Majority of the study participants were female (87.9%), who were first time mothers and aged 26–31 years (38.6%) with a primary level education; the practice of CBE was high among the less educated which is different from the findings of [15] where the highly educated had practiced CBE the more. However, the former finding is in line with [16] where (36.2%), were Catholics, (39.6%) married, (76.2%); 63.1% were employed and 56.7% had two or more children below 5 years old. The highest proportion of the respondents were from the central region 95, (31.9%).

### Proportion of households that practiced CBE

The proportion of households that practiced CBE was high at 269 (90.3%) which is higher than what is reflected in other sources [1]. The caregivers of the study participants were further asked where they sought the canine bud extraction services and a majority 69.8% mentioned that they had gone to traditional healers; 12.1% of the study participants had had their children’s canine buds extracted by a health worker. The children had their teeth extracted at 3 years 48%, 2 years 33.2%, 4 years 12.4%, 1 year 6% and 5 years 0.3%. With regards to instruments used during CBE, 82 (27.5%) reported that bicycle spokes were used, 71 (23.8%) said that surgical/razor blades were used, 54 (18.1%) mentioned nails, and 33 (11.1%) mentioned knives; 19.5% did not know what had been used during the CBE process, 56.9% of these had gone to traditional healers.

### Knowledge

The study revealed that 94.3% of the mothers know that CBE treats diarrhea [OR = 7.5 (2.0–27.6)]. These results are similar with that of a study that was conducted in Ethiopia where 68.7% of the study participants said that diarrhea was the major cause of canine tooth bud removal [17] and in a cross-sectional study in North West Ethiopia where 84.5% of mothers preferred canine tooth bud removal for the treatment of diarrhea [3]. This could be due to poor attitudes as reflected in another study in Kenya which revealed that the Maasai knew that diarrhea causing diseases were brought about by the canine tooth buds, despite having received sensitization interventions jointly run by the University of Nairobi, Kenya Medical Research Institute and the Kenya Medical Training College, among others [18]. This may be due to poor attitudes towards the practice of extraction of milk teeth.

The study revealed that 92.3% of the mothers know that CBE is the traditional treatment for fever [OR = 17.1 (4.7–61.6)]. Dr. Graham et al., [19] the study also revealed that there are strong cultural beliefs that the swelling in the area of the gums is associated with un-erupted cuspids which is the cause of persistent fever.

It should not be a surprise in the unindustrialized world to associate disease conditions that cause fever to be teething since febrile conditions are among the leading causes of morbidity and mortality, and hence subject their infants to oral mutilation techniques. In this regard, the basis for canine bud extraction would be presumed alleviation of fever among the young children [12] this was related to a study by Graham et al. [19] among household heads in western Uganda (Bushenyi district) where the cause of false teeth was mainly attributed to natural causes such as excessive or prolonged fever rather than to supernatural causes [12] .These findings suggest that canine bud extraction is still being practiced, which reflects ignorance of, and/or a negative reaction towards the nationally and globally recommended Western allopathic medicine for good oral health.

The study also revealed that 62.4% of the mothers know that CBE treats malaria [OR = 4.8 (1.4–17.3)]. Other findings suggest that education on simple hygiene will counteract some infectious conditions such as malaria, and that families with a good understanding of the causes of malaria could foresee that the relatively rough traditional practices might become less attractive [20]. Malaria being the leading cause of morbidity and mortality in Nakawa division, the children’s risks to undergo these procedures are extensive and may stretch from septicemia and probable HIV transmission to potential death. There is, therefore, a need for precise strategies concerning management of suspected cases within Nakawa as a suburb of Kampala capital city.

### Cultural beliefs and CBE

With the era of globalization people move from one part of the world to another, immigrants are interested in cultural socialization while teaching their children about their ethnic customs and traditions [4] considered as a way of identification. Even for those who are educated they still regard traditional beliefs when it comes to ill health. With no exception this study showed that immigrants and people who were coming from Northern Uganda neighboring South Sudan were 4 times more likely to practice CBE compared to Western tribes and all participants from foreign tribes had practiced CBE, this is in line with previous studies [2].

More than half of the respondents reported that they had cultural beliefs related to false teeth, they believed that false teeth were caused by germs, caused by “stepping on false teeth when pregnant”, and that it was a family disease. On the other hand, some respondents still believed that it was a taboo practicing CBE in their culture saying, *“it is a foreign cultural practice”…and “it is associated with witchcraft”.*

However, no significant association was established between the socio-cultural factors like the cultural belief concerning the cause of “false teeth” and was rather seen to be caused by group influence since majority admitted being drawn to the practice by their neighbors. Tribe of the respondents and whether the practice was considered a taboo in the study respondents’ cultures was not one of the factors associated to the practice. Furthermore, the study findings also indicate that majority of the respondents perceive CBE as a good practice hence the high proportion of households practicing CBE observed.

The custom of canine bud removal has detrimental consequences on children’s general health and dental care [2] in that some children are reported never to have teeth grow in places where the canine bud was extracted. Parents of such children exposed to canine bud extraction are also emotionally traumatized especially if they did not consent to CBE, which was done on their children in the name of cultural norms by their relatives or in-laws.

## Conclusion

A refined approach in communication on dental health is required by professional dentists and the Ministry of health in educating parents, guardians and caregivers, as they are key players in upholding cultural norms and traditions. There is need to sensitize health workers particularly those it the lower cadres who may be more likely to practice quackery about the consequences of conducting CBE. The child’s rights should always be prioritized.

## Abbreviations

CBE: Canine bud extraction
DCBE: Deciduous canine bud extraction
IHSU-REC: International Health Sciences University-Research Ethics Committee
IOM: Infant oral mutilation
TBE: Traditional bud extraction
